# Crosstalk of injured podocytes with parietal epithelial cells through Wnt4/β-Catenin signaling

**DOI:** 10.1038/s41598-025-04092-3

**Published:** 2025-06-04

**Authors:** Eike Schwartze, Eva Pfister, Nicole Endlich, Tim Endlich, Kerstin Amann, Maike Büttner-Herold, Jeff Pippin, Stuart J. Shankland, Christoph Daniel

**Affiliations:** 1 Department of Nephropathology, Institute of Pathology, Friedrich-Alexander-University Erlangen-Nuremberg (FAU) and University Hospital, Krankenhausstr. 8-10, 91054 Erlangen, Germany; 2 Division of Nephrology, University of Washington, Seattle, WA USA; 3 Nipoka GmbH, Greifswald, Germany

**Keywords:** Podocyte, Injury, Parietal epithelial cell, Wnt-pathway, Crosstalk, Cell signalling, Mechanisms of disease, Cell biology, Nephrology, Kidney diseases

## Abstract

Focal segmental glomerular sclerosis (FSGS) is considered an irreversible lesion in kidney disease. Here, we investigated the role of the wnt4/β-Catenin signaling pathway in FSGS lesion formation and the crosstalk between PECs and podocytes in a transgenic FSGS rat model and human primary FSGS to explore potential sex-specific differences and therapeutic options. After model induction in rats, we observed strong podocytes loss on day 7, which was significantly higher in male than in female rats. Starting at d14, both glomerular mRNA and protein expression of Wnt4 were increased, but more pronounced in males. Wnt4 was localized to podocytes and β-Catenin to Pax8-positive lesions. The Wnt4 target gene CD44 was strongly upregulated on d7 and increased until the end of the experiment (d42). In cell culture, we confirmed that injured podocytes expressed and secreted Wnt4, which stimulated the expression of the Wnt target gene Axin2 in PECs but not in podocytes. Wnt4/β-Catenin pathway activation was confirmed in human biopsies with podocytopathic FSGS. In conclusion, the canonical Wnt/β-Catenin axis plays a critical role in the crosstalk between PECs and injured podocytes. Furthermore, sex-specific differences in podocyte injury and regeneration appear to be, at least in part, Wnt4-mediated.

## Introduction

The increasing incidence of chronic kidney disease (CKD) progressing to end-stage renal disease (ESRD) poses a major challenge to modern medicine and strains the financial resources of global healthcare systems^1^. Glomerulopathies such as podocytopathic focal segmental glomerulosclerosis (FSGS) are often underestimated in contrast to more common diseases (e.g. diabetic and hypertensive kidney disease) and have limited treatment options. Although secondary and hereditary forms have been described^2^, understanding the pathogenesis of this mostly idiopathic disease and the signaling pathways involved is crucial for the development of new therapies that will allow us to combat this disease^3^. It is generally believed that this disease is caused by injury to the podocytes. Podocytes are terminally differentiated and highly specific renal cells with foot processes that form the cellular part of the filtration barrier. Due to the limited regenerative capacity of these cells, podocyte loss is critical, as loss of more than 20% of podocytes leads to FSGS, synechiae, and expansion of the mesangial matrix^4^. This raises the question of what mechanisms are involved in the compensation of this podocyte loss and the formation of FSGS lesions. In response to podocyte loss, parietal epithelial cells (PECs) surrounding Bowman’s capsule have been shown to migrate into the glomerular tuft^5^. To migrate, PECs undergo an epithelial-to-mesenchymal transition (EMT) and some of these cells can differentiate into podocytes at their destination by mesenchymal-epithelial transition (MET)^6^. If MET does not occur, the migrated PECs develop a fibroblast-like phenotype that produces extracellular matrix^7^. The progression of FSGS lesions severely limits the filtration of the affected nephrons. Wilms’ tumor 1 (WT1) is a transcription factor involved in the development of the urogenital system and is particularly expressed in podocytes^8^. Recent studies have shown that WT1 activates the Wnt4/β-Catenin signaling pathway and is critical for nephrogenesis^9^.

The secreted glycoproteins of the Wnt family stabilize β-Catenin in the cytosol and prevent its degradation. After translocation to the nucleus, β-Catenin acts as an inducing transcription factor for proliferation genes, including CD44. β-Catenin is known to induce EMT^10^, which may be a consequence of CD44 effects in cell–cell and cell–matrix interactions^11^. Normally, Wnt4 secretion is reduced to a minimum after nephrogenesis in the kidney^12^. However, upon glomerular injury, Wnt4 expression is re-induced, leading to increased EMT and induction of β-Catenin, mimicking pathways known from nephrogenesis^13^. Notably, PECs require tight regulation of Wnt to develop an appropriate phenotype^14^. This suggests that Wnt expression has an important impact on the differentiation of PECs. Therefore, in our study, we investigated the potential Wnt4-mediated crosstalk between damaged podocytes and PECs in the formation of FSGS lesions.

In our study, using an established podocyte injury model in the rat^4^, we show that PECs migrate to the glomerular tuft after injury. This is associated with activation of the Wnt4/β-Catenin signaling pathway in this context and correlating with the extent of segmental sclerosis. Interestingly, we also found sex-specific differences in injury and signaling. The crosstalk was also investigated in in vitro experiments with podocytes and PECs. In a human cohort of podocytopathies, i.e. MCG and FSGS patients, we were able to demonstrate the relevance of the results in a clinical context.

## Materials and methods

### Podocyte injury model in the rat and assessment of glomerular disease

Transgenic rats (Fischer 344-Tg (DTR) C354Wig rats; RGD_ID1302921) were used expressing the human diphtheria toxin receptor (hDTR) exclusively in podocytes under control of the podocin promoter and were kindly provided by Prof. Roger C. Wiggins from University of Michigan and bred in the Preclinical Experimental Animal Center (PETZ) of the Medical Faculty of Friedrich-Alexander University Erlangen-Nürnberg^4^. Once injected with diphtheria toxin (DT) dose-dependent podocyte death is observed within the following seven days. The experimental protocol for the animal studies was approved by the German regional committee for animal care and use, which is equivalent to the US Institutional Animal Care and Use Committee and authorized by the responsible governmental department (Regierung von Unterfranken permit 55.2-2532-2-255). Animal experiments were described in accordance to the ARRIVE guidelines^15^ and all experimental methods were performed in accordance with the relevant guidelines.

**Fig. 1:**
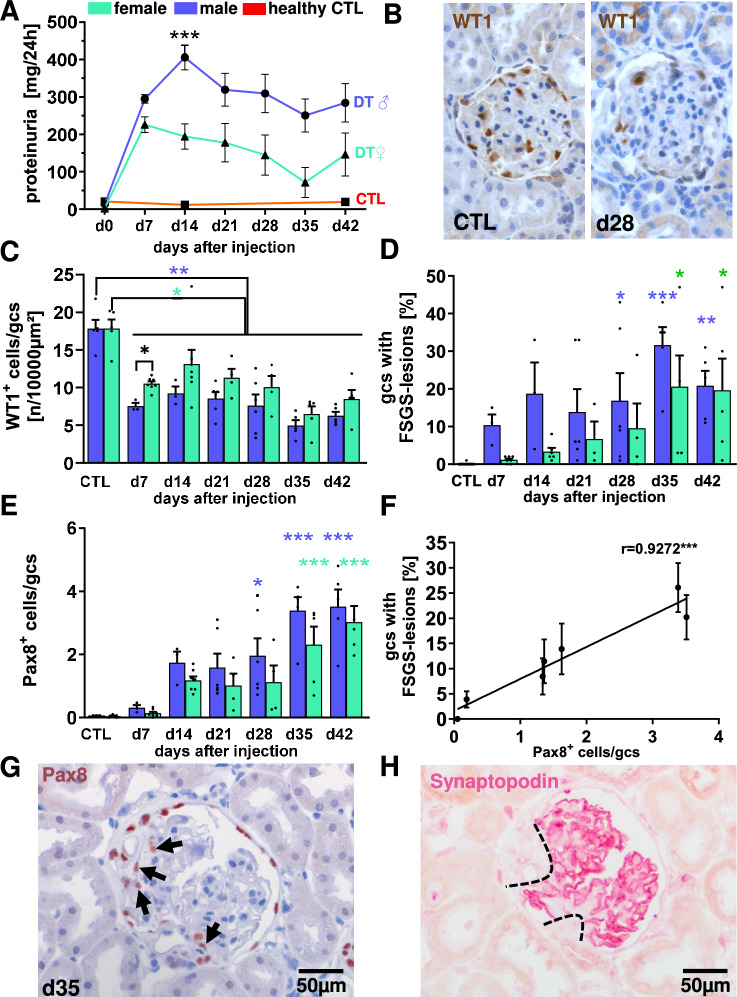
DT-induced podocyte loss increases proteinuria, FSGS lesions and PEC migration to the glomerular tuft. Proteinuria was assessed for rats using metabolic cages; DT injected rats grouped by sex vs. PBS injected rats (●male/▲female vs. ■controls) (**A**). Representative pictures of the WT1-staining in healthy control (CTL) and DT-treated rats on day 28 are shown (**B**). Depletion of WT1-positive cells per glomerular cross section (gcs) in DT-injected rats compared to controls over time (**C**). The percentage of FSGS-lesions in male and female rats, as assessed on PAS-stained sections, is shown for all time points (**D**). The number of Pax8-positive cells on the glomerular tuft per glomerular cross section was evaluated using immunohistochemistry (**E**). The percentage of FSGS-lesions correlate with Pax8-positive cells per gcs (**F**). Representative images of a glomerular section with FSGS lesion in a rat 35 days after DT injection stained by immunohistology for Pax8 (**G**, arrows) and the subsequent section with synaptopodin (**H**) with a dashed line to mark the FSGS lesion. *p < 0.05 **p < 0.01 ***p < 0.001; colored asterisks indicate significance within each sex.

Forty 12-week-old rats of both sexes were divided into two groups (30 experimental, 10 control) and maintained in a specific pathogen-free facility with standard chow (Sniff Spezialiäten GmbH, Soest, Germany) and ad libitum access to water. Female rats had significantly lower body weights compared to male rats (Fig. S1). In the experimental group, podocyte injury was induced by injection of 15 ng diphtheria toxin (DT)/kg body weight (Sigma Aldrich, Deisenhofen, Germany) diluted in 0.2% normal rat serum in phosphate- buffered saline (PBS, Sigma Aldrich Chemie GmbH) via the tail vein under isoflurane anesthesia using a maximum injection volume of 300 µl. The control group received PBS only. The experimental group was divided into 3 subgroups of 10 rats each (Fig. 1). The control group was divided into two groups of five rats each. Renal survival biopsies were taken one week before the end of the experiment at weeks 2, 4, and 6 to evaluate disease progression. For this purpose, the animals were administered Buprenovet (Bayer, Leverkusen, Germany) at a dose of 0.05 mg/kg body weight s.c. prior to surgery. After isoflurane (anesthesia, the left flank was shaved, disinfected with KODAN tincture forte (Schülke & Mayr GmbH, Norderstedt, Germany), the flank opened with a 1.5 cm incision, and the kidney carefully lifted out and fixed with 2 sterile swabs. The upper pole of the kidney was then resected with a scalpel and the cut surface closed with a collagen sponge (KOLLAGENresorb, Resorba medical GmbH, Nuremberg, Germany). Finally, the kidney was returned to its original position and the abdominal incision was closed with an absorbable suture and a continuous seam, and the skin was closed with single button suture (Ethicon-coated Vicryl 6–0, Johnson &Johnson International). Blood samples were collected for analysis of serum creatinine and urea levels as surrogate markers of renal function on the day of the survival biopsy and at the end of the experiment. For analysis of proteinuria, rats were placed in metabolic cages for 23 h prior to survival biopsy surgery and endpoint. Proteinuria was measured using the Bio-Rad Protein Assay (Bio-Rad Laboratories GmbH, Munich, Germany) and creatinine levels per liter of urine were determined for normalization. At the end of the experiment, rats were euthanized by exsanguination followed by perfusion under isoflurane anesthesia. For perfusion of anesthetized rats, the aorta was punctured with an indwelling catheter, perfused with 40% dextran supplemented with 0.2% procaine followed by 0.9% NaCl, and the vena cava was simultaneously opened.

### Isolation of glomeruli from rat kidneys and mRNA expression analysis

The partially resected kidney removed at the end of the experiment was minced with a razor blade. The homogenized kidney tissue was pressed through a 100 µm mesh cell sieve (Greiner Bio-One, Frickenhausen, Germany) using the plunger of a sterile 2 ml plastic syringe and continuously rinsed through the sieve with PBS containing 2% FCS (wash buffer). The eluate was then passed through a 70 µm sieve to remove smaller tissue fragments. Finally, the larger glomeruli retaining on sieve were transferred from surface to a reaction tube containing wash buffer and sedimented at 300 g for 5 min. Finally, the sediment was lysed in RLT buffer and total mRNA was isolated using a RNeasy Mini Kit (Qiagen, Hilden, Germany) according to the manufacturer’s instructions. Reverse transcription reactions and real-time PCR were performed using Power SYBR Green on a 7500 Fast Real-time PCR System (both Applied Biosystems, Weiterstadt, Germany) according to the manufacturer’s instructions. Real-time PCR data were analyzed using SDS v1.3 software (Applied Biosystems), and the relative expression of mRNA levels of target genes was calculated using the comparative delta Ct method^16^. Primers used in this study are listed in Supplemental Table 1.

### Immunohistochemistry and immunofluorescence staining

Kidney biopsies were fixed in 4% paraformaldehyde, dehydrated in series of alcohol and xylene and finally embedded into paraffin followed by cutting into 2 µm sections. After dewaxing in xylene und rehydration, endogenous peroxidase was blocked using 3% H_2_O_2_ (the latter step was omitted for immunofluorescence staining). For antigen retrieval sections were cooked for 2.5 min at 120 °C in target retrieval solution pH 6 (DAKO Deutschland GmbH, Hamburg, Germany) using a decloaking chamber (BIOCARE medical, Pacheco, CA, USA). Primary antibodies were diluted in 1% BSA/Tris-buffer incubated on the tissue for 60 min at 37 °C. Anti-WT1 and anti-Podocin were used to identify podocytes. Anti-Pax8 antibody was used to identify PECs^17^. Wnt4 and β-Catenin and CD44 as a wnt4 downstream target were stained for investigation of the wnt4/β-Catenin pathway. Detailed information on the used primary antibodies are listed in Supplemental Table 2. After washing steps, a biotinylated secondary antibody incubated for 30 min followed by ABC-Kit (Vector) and diaminobenzidine (ImmPACT) as a substrate were used to visualize biotinylated antibodies. Haematoxylin was used as a counterstain and sections were dehydrated in a graded alcohol series and covered with Eukitt (Sigma-Aldrich) as a mounting medium. For immunofluorescence double staining sections were incubated with fluorochrome-labled secondary antibodies for 30 min followed by covering the sections with TrueView mounting kit (Vector laboratories) and analyzed using laser scanning confocal microscopy (LSM Zeiss 710 and Zen software, Zeiss GmbH, Jena, Germany). Detailed information on the used secondary antibodies were listed in Supplemental Table 3.

### Quantification of glomerular changes

Per slide 50 glomeruli were analyzed and occurrence of segmental sclerosis with associated cellular proliferation and the number of Pax8-positive cells on the glomerular tuft were quantified by two independent investigators using a conventional Olympus Bx60 light microscope. In addition, percentage of positive staining area per total glomerular area was measured with ImageJ^18^ using the threshold tool to exactly determine the stained area in 50 manually circled glomeruli.

### Podocyte exact morphology measurement procedure (PEMP)

A structured illumination microscope N-SIM-S (Nikon, Japan) equipped with a 100 × immersion objective was used. PEMP was performed by NIPOKA GmbH using their standard protocol as previously described^19,20^.

### Human kidney tissue

For the human cohort biopsies were used form 22 patients with Minimal Change Disease (MCD), 19 patients with podocytopathic FSGS and 19 controls without signs of glomerular disease (11 acute tubular injury (ATI), 8 hereditary collagen IV-associated nephropathy). Biopsies were fixed in 4% formalin and handled as described above. The use of archival material of the Department of Nephropathology was approved by the Ethics Committee of the Friedrich-Alexander University of Erlangen-Nuremberg, waiving the need for retrospective informed consent for the use of archived rest material (Re.-No.4415 and Re. No.22–150-D). Human research was performed in accordance with the Declaration of Helsinki.

### Temperature sensitive mouse podocytes and parietal epithelial cells (PECs)

Conditionally immortalized and temperature-sensitive mouse podocytes were used for in vitro experiments. Using RPMI-1640 (Sigma-Aldrich, Deisenhofen, Germany, R8758) supplemented with 10% fetal bovine serum (FBS, Biochrom GmbH, Berlin, Germany, S0615), 1% penicillin/streptomycin (Sigma-Aldrich, P0781), 1% HEPES buffer (Sigma-Aldrich, H0887) and 0. 1 mM sodium pyruvate (Sigma-Aldrich, S8636) as growth media. Cells were cultured in Primaria flasks (Corning Incorporated-Life Sciences, Durham, NC, USA; #353,810) previously coated with rat collagen type 1 (Corning, Discovery Labware Inc, Bedford, MA, USA, #354,236). For growth-permissive proliferation, podocytes were cultured at 33 °C and 5% CO2. The medium was supplemented with 50 U/ml interferon gamma (IFN-γ, Sigma-Aldrich, I4777). After proliferation, podocytes were transferred to uncoated Primaria flasks and cultured under growth-restricted conditions (without IFN-γ) at 37 °C. At day 4, podocytes were plated on Primaria cell culture dishes at a density of 8000 cells/cm^2^. Experiments were performed on day 14. At this time, the cells were considered fully differentiated. Media were changed every other day under both conditions.

The same protocol was used for mouse PECs except that the FBS concentration was 2% in all growth media. Cells were replated at a concentration of 20,000/cm^2^ after 4 days of growth restriction.

### Podocyte injury in vitro model and evaluation

Differentiated podocytes were injured by treatment with puromycin amino nucleoside (PAN, Sigma Aldrich, P7130). 30 µg/ml PAN was added to the podocyte media, since this dose was shown to be effective in damaging podocytes in vitro in previous work^21^. After 6 h, the medium was changed back to growth-restrictive medium without PAN. After 24 or 48 h, the supernatant was collected. Real-time PCR analysis on the cells was performed to quantify the expression of podocin, Wnt4, β-Catenin, Axin2 and CD44 (primers are listed in the Supplementary Table). Wnt4 activity was verified in podocyte supernatants using a bioreporter assay^22^ (by Prof. Behrens, Department of Experimental Medicine II, Friedrich-Alexander University Erlangen-Nuremberg).

### Wnt/β-Catenin pathway stimulation in mouse PECs

To stimulate the Wnt4/β-Catenin pathway at a physiological level, the growth media of cultured mPECs was changed to the supernatant from the podocyte damage model. After an incubation period of 24 or 48 h, the expression of β-Catenin and CD44 was determined by real-time PCR. As a positive control, PECs were incubated with recombinant murine Wnt4 at 50 ng/ml (R&D Systems, Minneapolis, MN, USA, #475-WN). The Wnt pathway inhibitor murine dickkopf-related protein-1 (DKK-1, R&D Systems, #O54908) was used at a concentration of 100 ng/ml to block the Wnt4/β-Catenin pathway.

### Statistical analysis

All statistical analyses were conducted using GraphPad Prism 10 software (version 10.1.2 GraphPad Software Inc., San Diego, CA, USA). Outliers were identified using the ROUT (robust regression and outlier removal) method with Q = 1%, and normal distribution was assessed using the Kolmogorov–Smirnov test. For normally distributed data, differences were assessed using one-way analysis of variance (ANOVA), followed by Tukey`s multiple comparison test. For data not meeting the assumption of normality, differences were instead assessed relying on the Kruskal–Wallis test followed by Dunn’s multiple comparison test. The Pearson correlation test was used for correlation analysis. A p-value lower than 0.05 was considered statistically significant (*p < 0.05; **p < 0.01; ***p < 0.001; ****p < 0.0001). Data were presented as bar graphs with mean ± SEM and individual data points.

## Results

### Sex-specific podocyte injury and FSGS lesion formation after podocyte depletion in hDTR transgenic rats

**Fig. 2:**
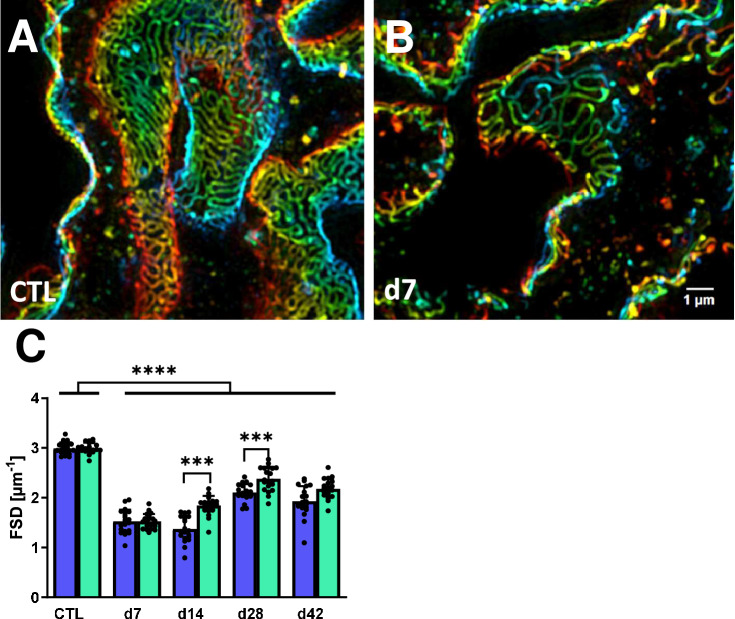
Changes in filtration slit densities after induction of DT-induced podocyte injury. Representative pictures of podocin and synaptopodin staining were used for FSD measurements. Podocin staining is shown for the healthy control (**A**) and 7 days after model induction (**B**). Evaluation of FDS using podocin staining and 3D-SIM super-resolution microscopy in paraffin sections from kidneys at different time points are shown (**C**). Blue bars representing male animals, green for females. ***p < 0.001; ****p < 0.0001.

First, we investigated the effects of intravenous injection of 15 ng DT/kg body weight on the development of proteinuria, podocyte damage, and FSGS lesion formation in rats expressing the human diphtheria toxin receptor under the podocin promoter. Before DT injection, the measured urine protein content was at a low level in all test animals regardless of sex (Fig. 1A, d0). Seven days after DT injection, proteinuria had increased significantly in rats of both sexes and had already reached a maximum in female rats. In male rats, proteinuria increased further to a mean of 400 mg/24 h on day 14 and was significantly and almost twice as high as in females at this time point (Fig. 1A, d14). Over the course of the experiment, the mean proteinuria decreased slightly and always showed a trend toward higher proteinuria in males than in females, although the significance level was not reached due to the relatively large variance (Fig. 1A). In addition, we assessed the glomerular filtration rate (GFR) as a measurement of kidney function, but found no significant changes between the time points and showing only a trend towards decreased levels in DT-injected rats (Fig. S2). The transcription factor WT1 is specifically expressed in the nucleus of podocytes (Fig. 1B). At all time points examined, the number of WT1-positive podocytes per glomerular section was significantly reduced in rats of both sexes (Fig. 1C). Only on day 7 after DT injection, the loss of podocytes was significantly greater in male than in female rats after DT injection (Fig. 1C, d7). Although no significant differences between the sexes were observed at other time points, the mean number of WT1-positive podocytes was always higher in females (Fig. 1C). The number of WT1-positive cells per glomerular area increased slightly in both sexes at day 14 and then showed a tendency of increased loss of these cells until the end of the experiment (Fig. 1C). While an average of 10% of the glomeruli in males show FSGS lesions on day 7 and more than 30% on day 35, the development of FSGS lesions in females was slower (Fig. 1D). Overall, however, the groups showed a high variance, so that a significant formation of FSGS lesions after DT injection could only be observed at the late time points. The epithelial marker Pax8 is also expressed by parietal epithelial cells (PECs). In parallel with the formation of FSGS lesions, the number of Pax8-positive PECs increased, and migrated to the glomerular tuft and participated in the formation of FSGS lesions in the area of the destroyed podocytes (Fig. 1E). This is also shown by the positive correlation (r = 0.927***) of glomeruli with FSGS lesions with the number of Pax8-positive cells on the tuft (Fig. 1F). In the neighboring sections it was seen that synaptopodin-positive podocytes were lost in areas with Pax8-positive cells on the tuft (Fig. 1G, H). To further assess podocyte morphology after injury, filtration slit density (FSD) was determined by podocin and synaptopodin staining followed by super-resolution microscopy 3D-SIM. While a uniform, dense distribution of slit diaphragms was observed in control animals (Fig. 2A), glomeruli from animals 7 days after DT injection showed an irregular, disrupted distribution (Fig. 2B). Compared to healthy controls, FSDs are significantly reduced in all rats after DT injection. On day 7 after model induction, FSD was lowest and comparable in both sexes. While FSD did not change in male rats at d14 compared to d7, it significantly recovered in females (Fig. 2C). Although a significant increase in FSD was also observed in males at d28, the increase was still significantly higher in females (Fig. 2C), indicating sex-specific differences in FSD recovery. At the last observation time point, d42, the FSD was very similar to d28, but the sex difference did not reach the significance level (Fig. 2C).

### Wnt4/β-Catenin pathway is involved in formation of FSGS lesions

Several cells are involved in the formation of FSGS lesions and PECs appear to be essential. We have shown that after podocyte loss, Pax8-positive PECs migrate from Bowman’s capsule to the glomerular tuft (Fig. 1). Therefore, we next investigated which signals damaged podocytes use to communicate with and activate PECs. We focused on the Wnt4/β-Catenin signaling pathway because it is known to be involved in the differentiation of mesenchymal cells into podocytes and PECs.

**Fig. 3:**
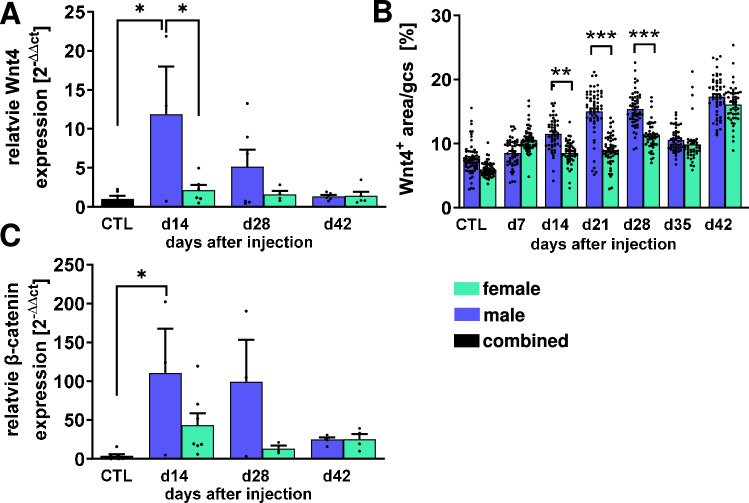
Wnt4/β-Catenin pathway activation is stronger in male rats after induction of podocyte injury. Glomerular Wnt4 mRNA expression, as assessed by Real time-PCR using isolated rat kidney glomeruli, was higher in male compared to female rats after induction of podocyte injury (**A**). Wnt4-positive glomerular area was analyzed using immunohistochemistry (**B**). Relative glomerular β-Catenin mRNA expression was measured using Real-time PCR (**C**). *p < 0.05 **p < 0.01 ***p < 0.001.

Relative Wnt4 mRNA expression was analyzed in glomerular extracts at two, four and six weeks and showed a significant increase (> tenfold) only in male rats two weeks after model induction (Fig. 3A), while the mean expression decreased at the following time points (Fig. 3A). The control group showed almost no Wnt4 mRNA expression, as this pathway is normally downregulated after differentiation^14^. In contrast, Wnt4 mRNA expression in female rats remained at a low level at all time points and was significantly different from that of male rats at day 14 (Fig. 3A). Indeed, by evaluating the immunohistochemical evidence for Wnt4 in the glomeruli, we confirmed that Wnt4 is upregulated at the protein level in the glomeruli of male animals after DT injection. Additionally, significantly more Wnt4 was present in the glomeruli of male rats than in female animals with podocyte damage at days 14, 21 and 28 (Fig. 3B). In contrast to the Wnt4 mRNA expression studies, increased Wnt4 was still detectable at the protein level in the glomeruli on day 42 after model induction (Fig. 3B). In the canonical Wnt signaling pathway, secreted Wnt4 binds to receptors on the surface of neighboring cells, consisting of a combination of frizzled proteins (Fz) and LRP5/6 coreceptors, and ultimately leads to the stabilization of β-Catenin, which then translocates to the nucleus and induces the transcription of Wnt target genes, which in many cases regulate cell proliferation and differentiation^23^. Therefore, we also examined the expression of the interaction partner β-Catenin. We found that β-Catenin was significantly increased in the glomeruli of male rats with podocyte injury only at d14, whereas glomerular mRNA expression in female rats was barely increased and not significant (Fig. 3C).

**Fig. 4:**
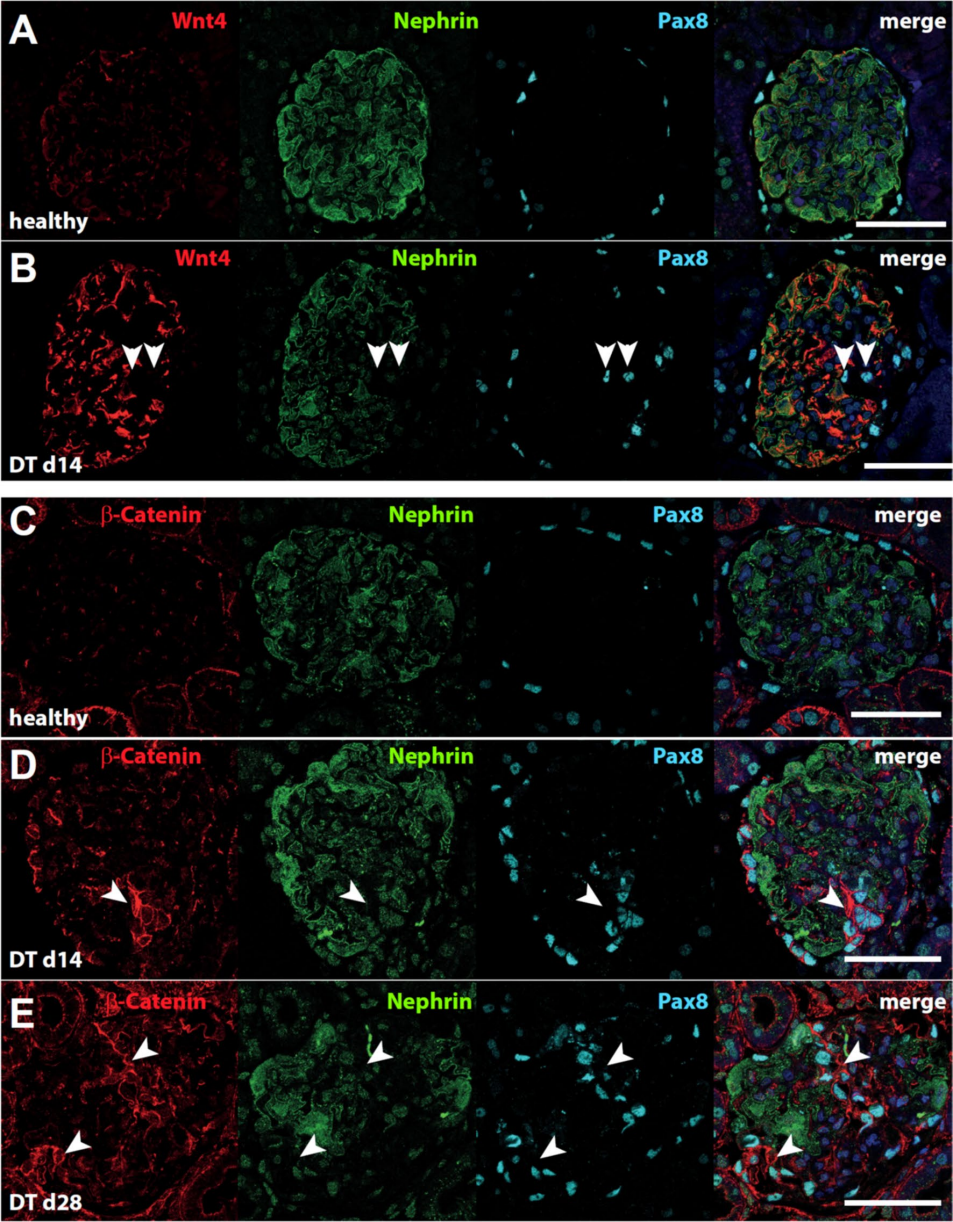
Colocalization of Wnt4 and β-Catenin in podocytes and PECs after induction podocyte injury. Immunofluorescence triple staining marking Wnt4 (**A**, **B**) or β-Catenin (**C**-**E**) in red, nephrin in green und Pax8 positive nuclei in blue. Colocalization of Wnt4 with nephrin and Pax8 was demonstrated in healthy rats (**A**) and on day 14 after DT-injection (**B**). Colocalization of β-Catenin with nephrin and Pax8 was demonstrated in healthy rats (**C**) and on day 14 (**D**) and on day 28 after DT-injection (**E**). In picture (**B**) arrowheads mark Pax8-positive PECs on the glomerular tuft, while in figure (**D**) and (**E**) arrowheads mark intense staining for β-Catenin in FSGS lesions. Scale bars represent 100 µm.

Co-localization studies with Wnt4, the podocyte marker nephrin and the PEC marker Pax8 showed that Wnt4 is barely expressed in healthy kidneys (Fig. 4A). On day 14 after model induction, Wnt4 was clearly detectable in podocytes, but not in areas where Pax8-positive PECs were detected on the glomerular tuft (Fig. 4B, arrowheads). β-Catenin is also barely detectable in the glomeruli of healthy rats (Fig. 4C). In rats with podocyte injury, β-Catenin colocalizes with Pax8-positive cells (Fig. 4D). A higher number of Pax8-positive cells on the glomerular tuft leads to increased β-Catenin detection in these cells (Fig. 4E). These data suggest that Wnt4 may be secreted by damaged or stressed podocytes and may lead to a stabilization of β-Catenin in PECs.

**Fig. 5:**
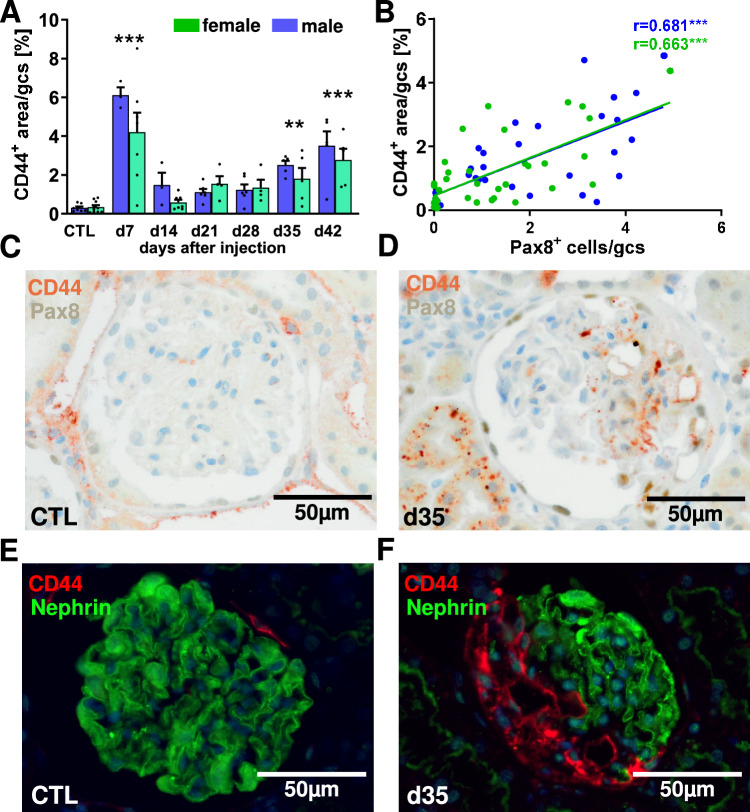
The Wnt4 target gene CD44 is expressed in Pax8-positive FSGS lesions. CD44-positive glomerular area was analyzed using immunohistochemistry (**A**) and correlated with the numbers of Pax8-positive cells per glomerular cross-section (gcs) using Pearson test, using the mean values of Pax8-positive cells and CD44-positive glomerular areas per glomerulus and including animals from all time points excluding day 7 (**B**). Representative immuno-double stains showing Pax8-positive cells (grey staining) and CD44-positive cells (red-brown staining) in healthy control rats (**C**) and 35 days after DT injection in hDTR-transgenic rats (**D**), while nephrin-positive cells did not express CD44 in controls (**E**) and 35 days after DT injection in hDTR-transgenic rats (F). **p < 0.01 ***p < 0.001.

We used immunohistology to stain CD44 as a Wnt4 target gene in the kidney sections. On day 7 after model induction, CD44 expression was highest, whereas hardly any CD44 was detectable in the glomeruli of healthy control animals (Fig. 5A, C). However, this early increase in glomerular CD44 expression does not appear to be due to renal cells but to the transient migration of leukocytes, which also express CD44 (Fig. S3, arrows). Already at d14, glomerular CD44 expression decreased sharply, but increased continuously with increasing duration of the experiment and reached the level of a significant increase at d35 and d42 compared to the healthy control (Fig. 5A, D). No significant difference in glomerular CD44 expression was observed between the sexes, but on average CD44 expression was higher in the glomeruli of male experimental animals at 4 time points (Fig. 5A). Excluding CD44 expression on day 7, which is heavily influenced by inflammatory cells and including all other time points, percent of glomerular CD44 area per glomerulus equally correlates with Pax8-positive cell number on the glomerular tuft per glomerulus in kidney sections from both male and female animals (Fig. 5B). Double staining for CD44 and nephrin showed, that glomerular CD44 expression was restricted to the lesion and was not expressed by nephrin-positive podocytes (Fig. 5E, F).

### Stressed podocytes secrete Wnt4 that can activate PECs in vitro

**Fig. 6:**
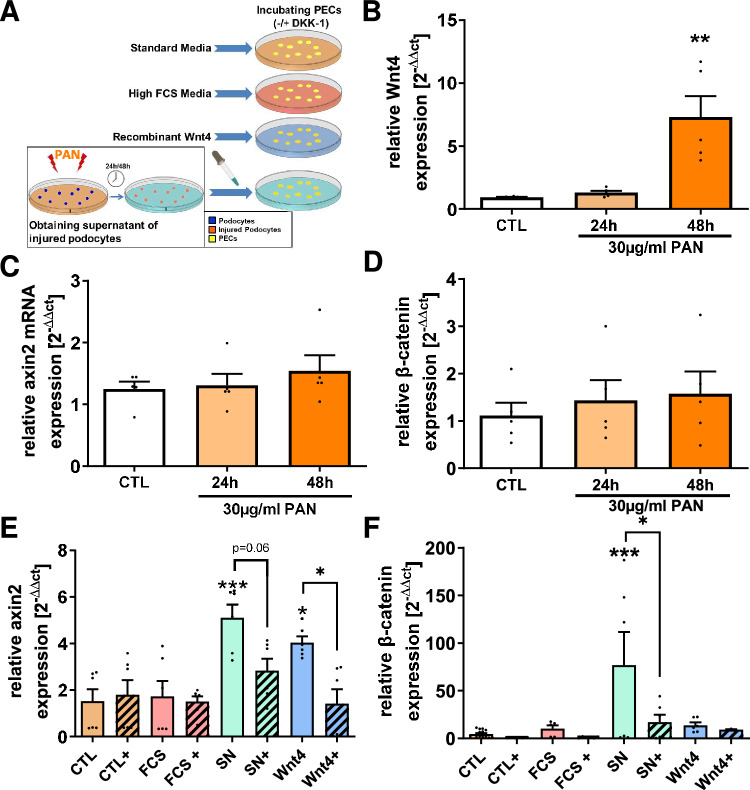
Wnt4 is secreted by injured podocytes that can elicit Wnt4 downstream targets in PECs. A schematic overview of the experimental setup of the in vitro experiments is shown (for details see M&M section) (**A**). mRNA expression of Wnt4 (**B**), axin2 (**C**) and β-Catenin (**D**) was assessed in cultured podocytes stimulated for 24 and 48 h with 30 µg puromycin amino nucleoside (PAN) compared to untreated controls (CTL). mRNA expression of Axin2 (**E**) and β-Catenin (**F**) was analyzed in mouse PECs incubated with PEC medium (CTL), podocyte medium (FCS), supernatant from PAN-stimulated podocytes (SN) or 50 ng/ml recombinant Wnt4 (Wnt4) with ( +) and without (unmarked) the addition of the Wnt inhibitor dickkopf-related protein 1 (DKK-1) using Real-time PCR. *p < 0.05 **p < 0.01 ***p < 0.001.

Having shown in vivo that Wnt4 is upregulated by podocytes in the injury model and that β-Catenin is stabilized on the glomerular tuft in Pax8-positive PECs, we wanted to investigate the Wnt4/β-Catenin-mediated crosstalk between injured podocytes and PECs. We used an in vitro model in which murine podocytes were transiently stressed with puromycin aminonucleoside (PAN) and then incubated for 24 and 48 h after a medium change (Fig. 6A). Wnt4 mRNA expression was significantly increased in the stressed podocytes 48 h after PAN stimulation (Fig. 6B), whereas relative Axin2, a highly specific Wnt target gene, and β-Catenin mRNA expression (Fig. 6C, D) as well as WT1 expression did not change in the podocytes (Fig. S4). The podocyte supernatant collected 48 h after PAN stimulation was used to incubate PECs in which the relative mRNA expression of the Wnt4 target gene Axin2 was subsequently measured by RT-PCR as a measure of Wnt4 stimulation. These supernatants were able to increase Axin2 mRNA expression by a factor of 5 compared to the control medium and thus comparable to the positive control with direct Wnt4 administration. Incubation with FCS had no stimulating effect. Wnt4 specificity was tested by adding the Wnt4 inhibitor DKK-1 (marked with + in Fig. 6E) and showed a significant reduction of Axin2 mRNA expression in PECs stimulated with Wnt4 and with podocyte supernatants, although the latter just missed the significance level (Fig. 6E). The Wnt4 present in the podocyte supernatant appeared not only to stabilize β-Catenin protein, but also to increase β-Catenin mRNA expression, which in turn was significantly reduced after the addition of DKK1 (Fig. 6F).

### PECs contributed to FSGS lesions in human biopsies and express β-Catenin

**Fig. 7:**
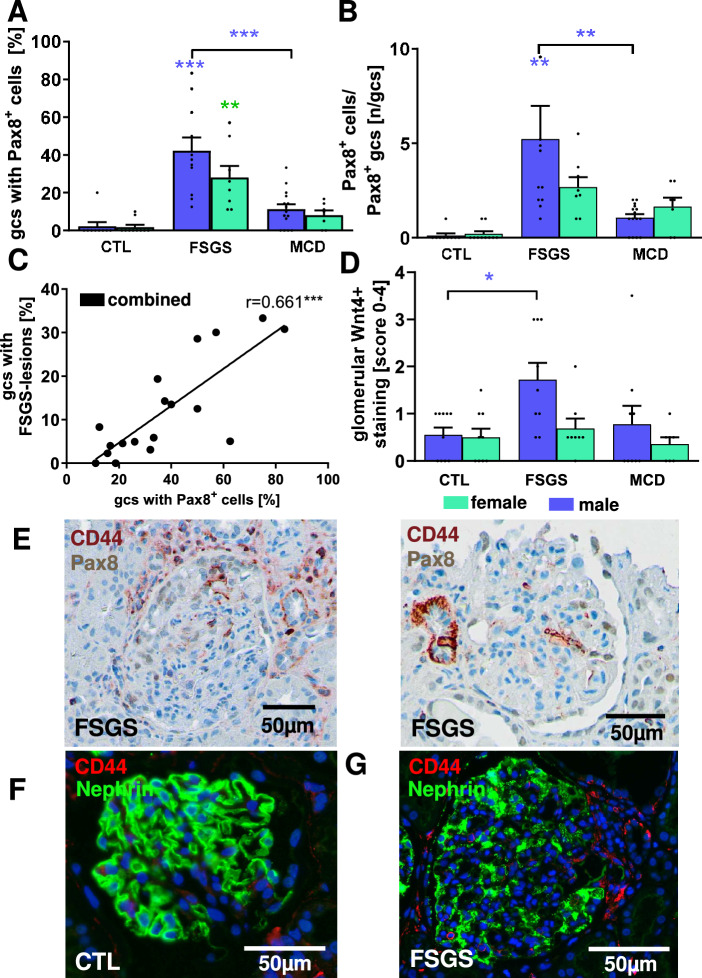
Wnt4 is expressed in glomeruli of human biopsies with primary FSGS. The percentage of glomerular cross-sections (gcs) with Pax8-positve cells on the glomerular tuft (**A**) and the numbers of Pax8-positive cells in gcs with Pax8-positive cells (**B**) were analyzed in human kidney biopsies from patients with acute tubular injury or hereditary collagen IV-associated disease (CTL, n = 19), minimal change glomerulonephritis (MCD, n = 22) and podocytopathic focal segmental glomerulosclerosis (FSGS, n = 19). Pax8-positive cells on glomerular tuft correlated positively with the percentage of glomeruli with FSGS lesion (**C**). Glomerular Wnt4-staining by immunohistochemistry was analyzed using a semi-quantitative score (**D**). In human kidney biopsies from patients with primary FSGS CD44 (brown staining) colocalized with Pax8 (grey staining) in glomerular cells using immuno-double staining (**E**). Double staining of CD44 (red staining) with nephrin (green staining) showed that CD44 was not expressed by nephrin-positive podocytes (**F**). *p < 0.05 **p < 0.01 ***p < 0.001; colored asterisks indicate significance within each sex.

Finally, we wanted to verify whether the mechanisms of FSGS lesion formation observed in the hDTR podocyte damage model could be reproduced in podocytopathic human FSGS. To this end, we first examined biopsies from male and female patients with primary FSGS for the presence of Pax8-positive cells on the glomerular tuft. Biopsies from patients with acute tubular injury and hereditary CollagenIV-associated nephropathy included together in a control group (controls) and minimal change disease (MCD) served for comparison. Of note, only glomeruli from the FSGS group showed a significant proportion of glomeruli with Pax8-positive cells on the tuft (Fig. 7A).

**Fig. 8:**
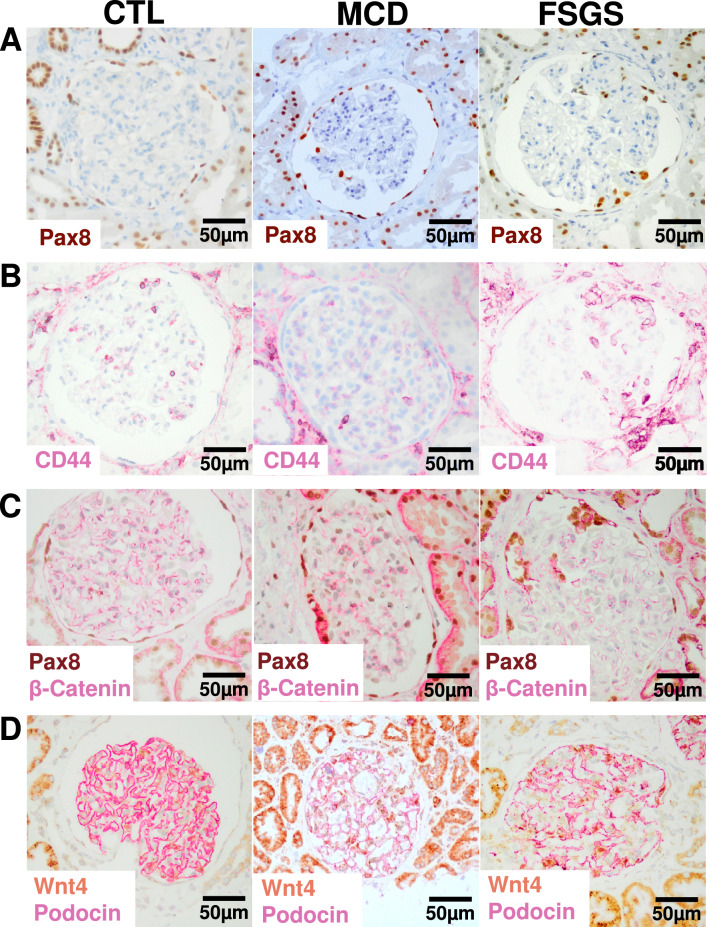
Immunohistochemistry for Pax8, CD44, β-Catenin, Podocin and Wnt4 in biopsies from healthy controls, MCD and primary FSGS. Representative pictures of glomeruli from healthy controls (CTL), patients with minimal change glomerulonephritis (MCD) and primary focal segmental glomerulosclerotic biopsies (FSGS) were stained for Pax8 (**A**, brown staining), CD44 (**B**, pink staining), double stained for Pax8 and β-Catenin (**C**, Pax8 brown and β-Catenin pink staining) and double stained for Wnt4 (brown staining) and podocin (pink staining) (**D**).

The number of Pax8-positive cells on the glomerular tuft was also significantly increased only in the FSGS group compared with the control group and the MCD group (Figs. 7A, 8A). The comparison of male and female patients with FSGS showed no significant difference in Pax8-positive cells, but especially the number of Pax8-positive cells per glomerular section tended to be higher in male patients with FSGS and showed no significant increase in female patients compared to controls (Fig. 7B). There are virtually no Pax8-positive cells on the glomerular tuft in patients with acute tubular injury and hereditary nephropathy, and very few in patients with MCD (Fig. 7A, B). In the glomeruli of FSGS patients, as in the hDTR animal model, the percentage of glomeruli with FSGS lesions correlates with the percentage of glomerular sections containing Pax8-positive cells (Fig. 7C). Glomerular Wnt4 expression was also significantly increased exclusively in the glomeruli of male patients compared to controls (Fig. 7D). Representative images of double staining for Pax8 and CD44 show that CD44 was also expressed by Pax8-positive cells in the glomerulus in human FSGS (Fig. 7E) and not by nephrin-positive podocytes (Fig. 7F), whereas in controls and MCD only single inflammatory cells usually expressed CD44 (Fig. 8B). β-Catenin was also clearly stained in human Pax8-positive FSGS lesions (Fig. 8C) as reproduced in our animal model (Fig. 4D-E, arrow heads) and was barely detectable on the tuft of both control groups (Fig. 8C). Wnt4 was less clearly detectable in the glomeruli of human kidney biopsies than in the animal model, but showed the most pronounced podocytic staining in patients with FSGS (Fig. 8D). These findings suggest that crosstalk of podocytes with PECs via the Wnt4/β-Catenin pathway may also occur in the genesis of FSGS lesions in humans.

## Discussion

The Columbia classification of FSGS differentiates five types one of which defines tip lesions as a diagnostic criterion^24^. Common to all forms is the interaction of damaged podocytes with PECs. Although other cells such as macrophages are also involved in the formation of crescents, the FSGS lesion is primarily formed by proliferating PECs^25^. Nagata et al. described that segmental sclerosis is most likely caused by these migrating PECs to combat leakage after podocyte loss^26^ correlating well with our histological findings. In our study, we were able to show that in the podocyte depletion animal model, Pax8-positive PECs over time migrate to and proliferate on the glomerular tuft after podocyte loss. However, the triggers that lead to PEC activation are poorly understood. CD44 is a surface molecule critical for inflammatory responses^27^ and has also been described as an activation marker of parietal epithelial cells which is crucial in crescent formation^28^. In this study, we were able to identify CD44-positive PECs on the glomerular tuft in both human podocyopathic FSGS and an animal model of podocyte injury, similar to others ^29–31^. The number of Pax8-positive PECs on the glomerular tuft correlated significant with the number of CD44-positive cells on the tuft and FSGS lesion formation. Although CD44 can be activated by other signaling cascades such as MAP kinases^32^, this molecule is also induced by the Wnt pathway^33^. Therefore, we focused on the canonical Wnt/β-Catenin pathway in the process of segmental sclerosis formation and associated PEC proliferation. Wnt4 is important in kidney development and here involved in branching of the ureteric bud but is downregulated to a minimum after nephrogenesis was completed^12^. In the glomeruli of healthy kidneys, we could not detect Wnt4 by immunohistology in either rats or humans. However, Wnt4 was clearly expressed in podocytes in the rat hDTR model and in biopsies from patients with FSGS. β-Catenin was upregulated in activated PECs, but not in podocytes. The Wnt/β-Catenin pathway has also been activated in other models of podocyte damage, such as the Adriamycin model, and has been implicated in disease development^34^. In this Adriamycin model, various Wnt molecules, including Wnt4, were upregulated in glomeruli and Wnt1 was localized in diseased podocytes, but β-Catenin was also activated in podocytes and not in PECs as in our study^34^. However, the Adriamycin model does not produce pronounced FSGS lesions. Our in vitro experiments clearly showed that although Wnt4 is expressed and secreted by podocytes, the expression of the Wnt4 target gene Axin2 was only detected in the PECs and not in the podocytes themselves. These data suggest, that Wnt4 may act as an activation signal for PECs when podocytes are injured. The significance of the Wnt/β-Catenin pathway in FSGS has also been demonstrated in other studies. Podocyte RNA sequencing showed that Wnt and ECM-associated genes play a central role in FSGS^35^. Furthermore, recent studies of a genetic form of FSGS with a mutation in the TRPC6 gene have also shown that this gene is significantly involved in the pathogenesis of FSGS via dysregulation of the Wnt and Hippo pathways^36^. In various kidney diseases, dysregulation of the Wnt pathway has been shown to lead to fibrosis and chronic changes^33,37–39^. Wnt (β-Catenin) may also promote further chronic changes via TGF-β, as Wnt signaling has been shown to damage podocytes via TGF-β^40^.

The association of lower Wnt/β-Catenin pathway activation with lower expression of kidney injury observed in our study makes this pathway a potential target for specific therapy. Many other studies have shown that the Wnt pathway plays a role in AKI and CKD. In our in vitro model, we have already successfully inhibited the Wnt/β-Catenin axis in PECs, effectively interfering with the interaction of PECs with podocytes. While DKK1 leads to a partial reduction of albuminuria in TGF-ß1 overexpressing mice^40^, it has been described that unbalanced activity of the pathway in both directions can lead to glomerular damage^23^. While transient upregulation of the Wnt4/β-Catenin pathway is protective in AKI, sustained Wnt4 activation accelerated AKI to CKD progression with fibrosis induction^39^. This indicates that the regulation of the Wnt/β-Catenin pathway must be adjusted depending on the stage and severity of the disease in order to avoid undesirable side effects. There are several compounds interfering with the Wnt/β-Catenin pathway tested in preclinical studies for treatment of CKD summarized in^41^, for example the Wnt/β-Catenin blocker geraniol^42^, prorenin receptor antagonist PRO-20^43^ or pachymic acid that upregulates Wnt signaling^44^. The Wnt/β-Catenin pathway is a relevant pathway in the pathogenesis of kidney disease, but also has important functions in kidney development and repair. Therefore, further extensive research is needed to identify safe molecules with suitable metabolism that can be used for treatment of kidney diseases in clinical trials.

In the hDTR podocyte injury model, in female rats we found less activation of the Wnt/β-Catenin pathway, which has been identified as a cause of increased renal injury and proteinuria in other models of renal injury^45^, compared to male animals. The increased activation of the Wnt pathway is probably not frankly sex-dependent, but rather a consequence of the greater damage in male rats.

Although all experimental animals received the same body weight-adjusted toxin dose in our rat model, podocyte loss after DT injection was significantly greater in males than in females on day 7 and remained elevated at later time points, although it did not reach significance. Proteinuria in females also never reached the level of proteinuria in males. Several sex-specific differences could account for this and sex hormones are the most important. Estrogen increases the expression of nephrin and Akt which might stabilize podocytes and make them more resistant to stress^46^. The protective role of estrogen in prevention of CKD was also suggested in a human prospective study reporting a higher hazard ratio of CKD incidence in women with low endogenous estrogen levels^47^. Gender differences are not limited to the animal model, but also occur in human renal disease. In our human cohort, the mean number of Pax8-positive PECs and Wnt4 expression were lower in female patients but did not reach significance due to the small number of cases. In a study with more patients, female patients with FSGS had a better outcome with less proteinuria^48^. The prevalence of ESRD is also lower in adult premenopausal women than in men in the USA^49^.

In conclusion, our study investigated the potential role of the Wnt/β-Catenin signaling pathway in the development of FSGS lesions. Podocyte depletion in the established hDTR animal model^4^ resulted in increased Wnt4 expression in the remaining podocytes. Secreted Wnt4 stabilizes β-Catenin in PECs and activated downstream protein production (Axin2, CD44) via this pathway, resulting in a proliferative phenotype that produced extracellular matrix and may promote FSGS lesion formation. Our findings may lead to identification of new targets for the therapy of this disease for which no specific treatments are currently available.

## Supplementary Information

Supplementary Information.

## Data Availability

The corresponding author will make the raw data supporting the conclusions of this article available without undue reservation upon request.

## Abbreviations

ATN: Acute tubular necrosis
CKD: Chronic kidney disease
CTL: Control group
DT: Diphtheria toxin
DKK-1: Dickkopf-related protein 1
EMT: Epithelial to mesenchymal transition
ESRD: End stage renal disease
FCS: Fetal calf Serum
FSD: Filtration slit density
FSGS: Focal segmental glomerular sclerosis
GFR: Glomerular filtration rate
gcs: Glomerular cross section
glom: Glomerulus
hDTR: Human diphtheria toxin receptor
IFN-gamma: Interferon gamma
MET: Mesenchymal to epithelial transition
MCD: Minimal change disease
PAN: Puromycin amino nucleoside
PAS: Periodic acid–Schiff
PBS: Phosphate-buffered saline
PEC(s): Parietal epithelial cell(s)
PEMP: Podocyte exact morphology measurement procedure
s.c.: Subcutaneous
SIM: Structured illumination microscope
SN: Supernatant
TF: Transcription factor
