# Supplementary material for: Crosstalk of injured podocytes with parietal epithelial cells through Wnt4/β-Catenin signaling
Source: Sci Rep. 2025 Jun 4;15:19652. doi: 10.1038/s41598-025-04092-3 (PMC12137821; doi:10.1038/s41598-025-04092-3)
Supplement: Supplementary file 1 — Supplementary Information. [file 41598_2025_4092_MOESM1_ESM.docx]

Supplement

**Crosstalk of injured podocytes with parietal epithelial cells**

**through Wnt4/ß-Catenin signaling**

**Eike Schwartze^1^, Eva Pfister^1^, Nicole Endlich^3^, Tim Endlich^3^, Kerstin Amann^1^,
Maike Büttner-Herold^1^, Jeff Pippin^2^, Stuart Shankland^2^ and Christoph Daniel^1^**

^1^Department of Nephropathology, Institute of Pathology, Friedrich-Alexander-University Erlangen-Nuremberg (FAU) and University Hospital, Erlangen, Germany;
^2^Division of Nephrology, University of Washington, Seattle, WA, USA; ^3^Nipoka GmbH

**Supplemental table 1: Primersequences, targets, and source (if not designed for this study*) used for quantitative real-time PCR.**

| **Target** | **Use** | **Sequence** | **Citation** |
| --- | --- | --- | --- |
| 18s f | Ribosomal RNA used as reference gene | TTGATTAAGTCCCTGCCCTTTGT | * |
| 18s r |  | CGATCCGAGGGCCTCACTA |  |
| WNT4 f | WNT4 | CTGGAGAAGTGTGGCTGTGA | * |
| WNT4 r |  | GGACTGTGAGAAGGCTACGC |  |
| WT1 f | podocyte marker | AGGACTGCGAGAGAAGGTTTTCT | * |
| WT1 r |  | TGGAATGGTTTCACACCTGTGT |  |
| CD44 f | Wnt/ß-Catenin pathway activation | CGACCCTTTTCCAGAGGCTACTAG | Stevens JW, Kurriger GL, Carter AS, Maynard JA. CD44 expression in the developing and growing rat intervertebral disc. Dev Dyn. 2000 Nov;219(3):381-90. doi: 10.1002/1097-0177(2000)9999:9999<::AID-DVDY1060>3.0.CO;2-P. PMID: 11066094. |
| CD44 r |  | TGGGTTCAATCGGGACCAAGAG |  |
| Ctnnb1 f  (ß-Catenin) | membranouse, cytoplasmatic and nucleic ß-Catenenin | TGAAGGTGCTGTCTGTCTGC | Damien P. Hewitt, Peter J. Mark, Arun M. Dharmarajan, Brendan J. Waddell, Placental Expression of Secreted Frizzled Related Protein-4 in the Rat and the Impact of Glucocorticoid-Induced Fetal and Placental Growth Restriction, Biology of Reproduction, Volume 75, Issue 1, 1 July 2006, Pages 75–81, https://doi.org/10.1095/biolreprod.105.047647 |
| Ctnnb1 r  (ß-Catenin) |  | GCTGCACTAGAGTCCCAAGG |  |
| Axin2 f | Wnt/ß-Catenin pathway activation | CAGGACCCACATCCTTCT | Jho, E.-h., et al., *Wnt/beta-catenin/Tcf signaling induces the transcription of Axin2, a negative regulator of the signaling pathway.* Molecular and cellular biology, 2002. **22**(4): p. 1172-1183. |
| Axin2 r |  | ACGCGGAGGTGCACGCGG |  |

**Supplemental table 2: Primary antibodies with information on host, appropriate fixative and supplier used in this study.**

| **Antigen** | **Host** | **Dilution** | **Fixative human** | **Fixative**  **rat** | **RRID** | **Supplier** |
| --- | --- | --- | --- | --- | --- | --- |
| Pax8 | pc rabbit | 1:50 | Fo | Zn, Fo | Not registred | Cell Marque, Rocklin, CA, USA |
| WT1 | pc rabbit | 1:200 | Fo | Fo | AB_722381 | Neo markers/ Lab vision/Thermo Fisher, Fremont, CA, USA |
| Podocin | pc rabbit | 1:1000 | Fo | Fo | AB_261982 | Sigma-Aldrich, St. Louis, Mo, USA |
| Beta-Catenin | mc mouse | 1:50 | Fo | Zn, Fo | AB_397555 | BD Biosciences, Heidelberg, Germany |
| WNT-4 | mc mouse | 1:2000 | Fo | Fo | AB_10986273 | Santa Cruz biotechnology, Santa Cruz, CA, USA |
| Nephrin | pc guinea pig | 1:500 | Fo | Zn, Fo | AB_1005584 | OriGene Tech, Rockville, USA |
| CD44 | pc rabbit | 1:100 | Fo | Fo | AB_2847859 | Abcam, Cambridge, UK |
| CD44 (human) | mc mouse | 1:50 | Fo | Zn | AB_2076596 | Dako/Agilent, Santa Clara, CA, USA |
| Synaptopodin | mc mouse | 1:50 | Fo | Fo | AB_1007637  Acris-Antibody (same clone) | Progen, Biotechnik GmbH, Heidelberg, Germany |

Pc, polyclonal; mc, monoclonal; Fo, formalin-fixed; Zn, zink-fixed

**Supplemental table 3: Secondary antibodies with information on host, type of application and supplier used in this study.**

| **Antigen** | **Host** | **Konjugation** | **Dilution** | **Application** | **RRID** | **Supplier** |
| --- | --- | --- | --- | --- | --- | --- |
| rabbit IgG | goat | biotin | 1:500 | IHC | AB_2313606 | Vector Laboratories, Burlingame, CA, USA |
| mouse IgG | horse | biotin | 1:500 | IHC | AB_2313581 | Vector Laboratories, Burlingame, CA, USA |
| mouse IgG | goat | Alexa Fluor 568 | 1:200 | IF | AB_2534072 | Thermo Fisher Scientific, Waltham, MA, USA |
| rabbit IgG | donkey | Alexa Fluor 488 | 1:200 | IF | AB_2535792 | Thermo Fisher Scientific, Waltham, MA, USA |

IHC, immunohistochemistry; IF, immunofluorescence microscopy

**Supplemental table 4: Gender distribution in the different experimental groups of hDTR rat model.**

| **Group** | **Females [n]** | **Males [n]** |
| --- | --- | --- |
| Control d14 | 0 | 5 |
| Control d42 | 0 | 5 |
| DT d14 | 7 | 3 |
| DT d28 | 4 | 6 |
| DT d42 | 5 | 5 |

DT, diphtheria toxin-treated

**Figure S1: Body weight of rats (12 weeks old) at the start of the experiment, separated by sex.** F= female; M= male.

**Figure S2: Glomerular filtration rate (GFR) after diphtheria toxin (DT) injection in transgenic rats expressing hDTR.** In both sexes, GFR initially decreased after DT injection. While male rats (blue line) are only able to recover slightly from this loss, females (green line) even increase their mean GFR in an earlier and stronger recovery period up to d21. Mean GFR values including both sexes are shown in black.


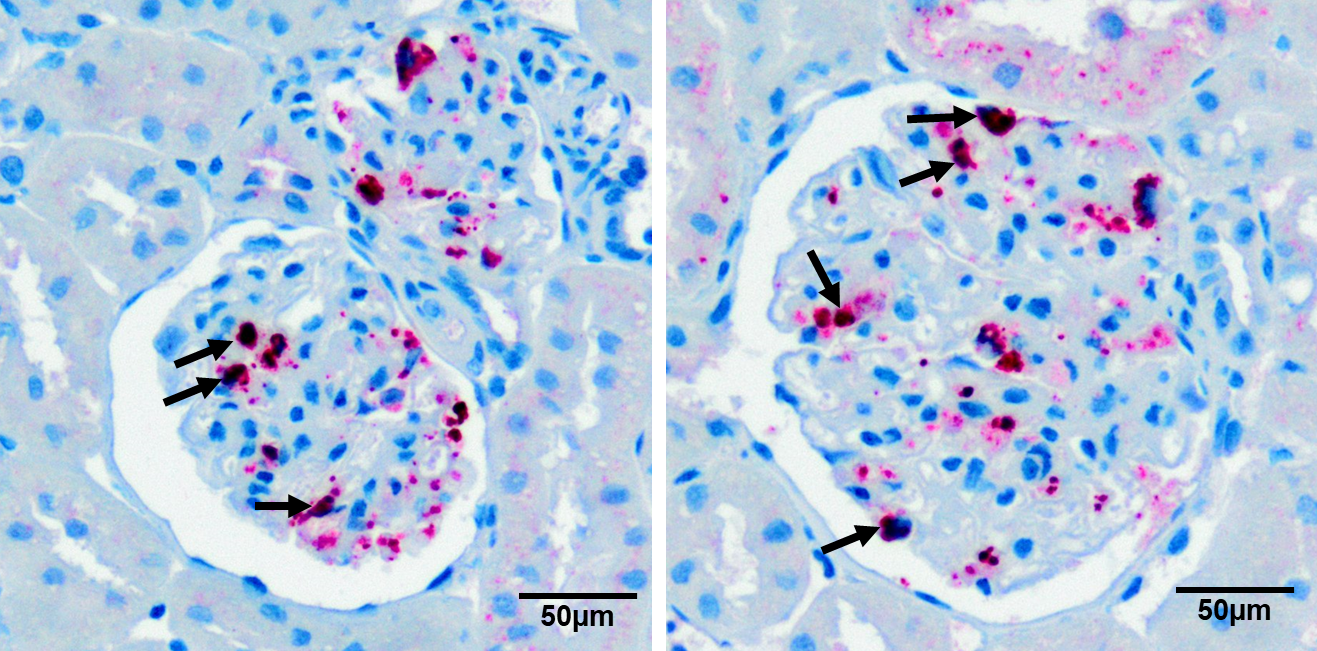


**Figure S3: Glomerular CD44 staining in the hDTR rat model seven days after DT injection.** Representative photographs show glomerular CD44-positive stain (red) on d7 after model induction as assessed by immunohistochemistry. Examples of CD44-positive immune cells are indicated by arrows.

**Figure S4: WT1 mRNA expression in cultivated podocytes after treatment with puromycin aminonucleoside (PAN).** WT1 mRNA expression in controls and 24h and 48 after stimulation with PAN was investigated using real-time PCR.
